# Supplementary material for: Evaluation of the Cholesterol-Lowering Mechanism of Enterococcus faecium Strain 132 and Lactobacillus paracasei Strain 201 in Hypercholesterolemia Rats
Source: Nutrients. 2021 Jun 9;13(6):1982. doi: 10.3390/nu13061982 (PMC8228983; doi:10.3390/nu13061982)
Supplement: Supplementary file 1 [file nutrients-13-01982-s001.zip › nutrients-1243925-supplementary.pdf]

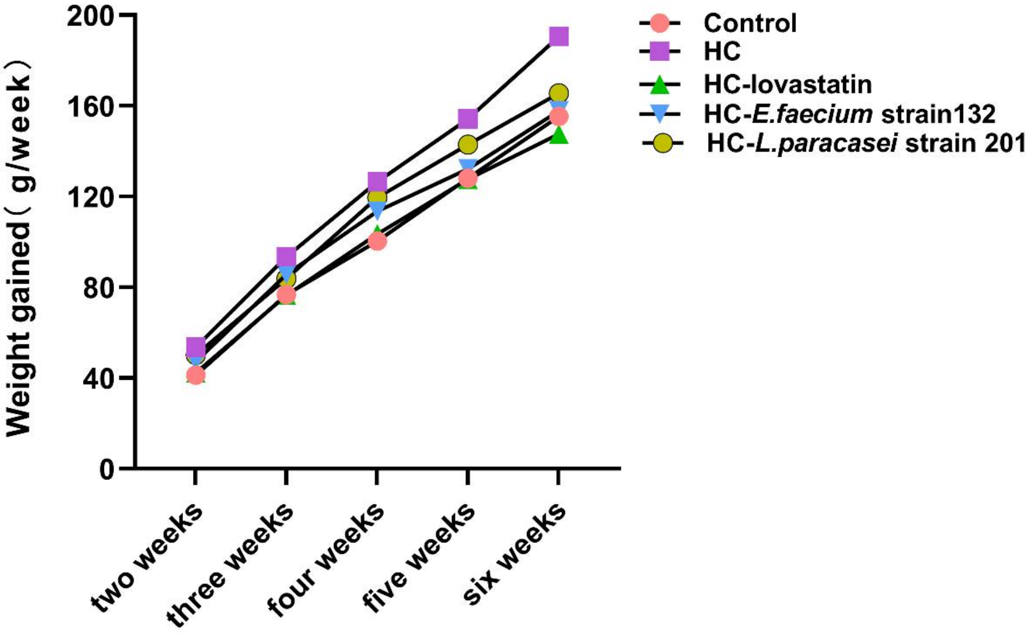

Figure S1 The trend of weight gained in each group at 6 weeks. Pink bar represents Control group, purple bar represents HC group, green bar represents HC-lovastatin group, blue bar represents HC-*E. faecium* strain 132 group, yellowish brown bar represents HC-*L. paracasei* strain 201group.

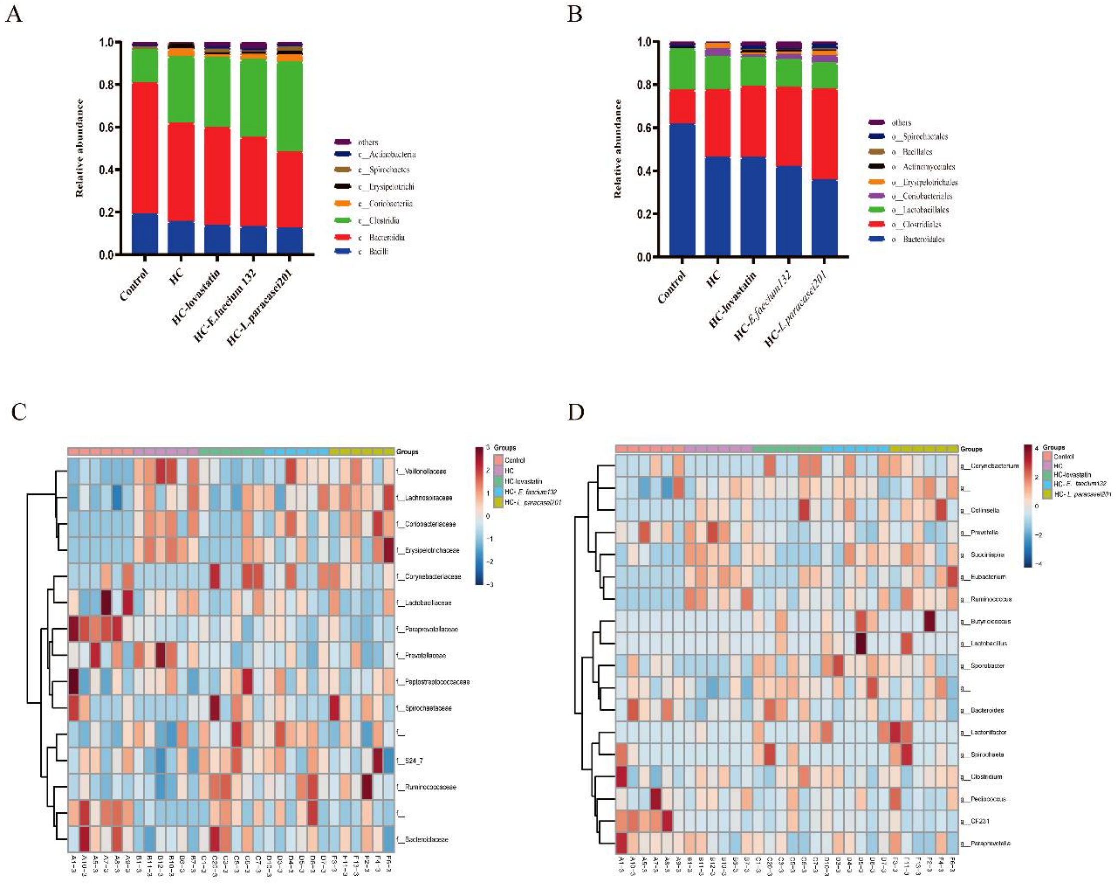

Figure S2 The species abundance of phylum, class, order, family and genus levels. (A) The relative abundance of class level. (B) The relative abundance of order level. (C) Heat map of family. (D) Heat map of genus.

Table S1 The cholesterol-lowing rate of isolates

serial number	strain number	lowering rate (%)	serial number	strain number	lowering rate (%)
1	0714-1	13.66±5.23	26	1328-3	17.25±0.51
2	0714-2	19.25±4.12	27	1337-5	17.24±2.34
3	0729-2	18.65±5.28	28	2056-2	16.75±4.73
4	0729-6	13.78±3.24	29	2056-3	13.28±4.24
5	0737-4	12.98±3.41	30	2060-5	19.25±1.61
6	0737-8	14.12±5.47	31	**2060-11**	**25.36±0.62**
7	1266-7	14.58±6.46	32	2065-1	13.78±2.65
8	1267-4	15.78±6.10	33	2065-4	10.02±1.54
9	1267-7	14.53±1.94	34	2065-7	17.30±2.57
10	1267-9	13.76±5.81	35	2065-8	16.54±7.47
11	1270-2	12.24±3.49	36	2074-2	16.53±4.32
12	1298-2	14.51±5.92	37	2074-5	15.58±4.90
13	1298-3	16.23±2.56	38	2074-6	16.89±7.92
14	1298-4	17.54±6.16	39	2074-8	13.24±6.12
15	1298-1	17.25±2.57	40	2074-9	13.53±8.15
16	1301-5	18.56±4.44	41	2076-5	11.57±5.03
17	1301-7	17.78±4.74	42	2076-7	14.45±5.49
18	1301-8	19.47±5.80	43	2076-8	11.36±3.37
19	**1302-1**	**23.62±6.73**	44	2077-2	15.30±1.67
20	1302-6	17.25±3.11	45	2077-3	13.27±7.35
21	1311-3	11.52±3.74	46	2077-5	10.35±.5.17
22	1311-4	10.20±0.91	47	2096-3	12.34±1.77
23	1326-1	17.12±5.64	48	2116-9	15.25±9.84
24	1328-1	10.25±4.51	49-107	others	<10.00
25	1328-2	16.27±2.46			
